# Needs and Preferences of Swedish Young Adults for a Digital App Promoting Mental Health Literacy, Occupational Balance, and Peer Support: Qualitative Interview Study

**DOI:** 10.2196/71563

**Published:** 2025-05-23

**Authors:** Martin Karaba Bäckström, Sonya Girdler, Ben Milbourn, Annika Lexén

**Affiliations:** 1 Mental Health, Activity and Participation Faculty of Medicine Lund University Lund Sweden; 2 Curtin Autism Research Group (CARG) Curtin University Perth Australia; 3 Center of Neurodevelopmental Disorders (KIND) Karolinska Institutet Stockholm Sweden; 4 Stockholm Health Care Services Stockholm County Council Stockholm Sweden; 5 School of Allied Health University of Western Australia Perth Australia; 6 Curtin School of Allied Health Perth Australia

**Keywords:** emerging adulthood, design thinking, mental health promotion, mental ill-health, needs assessment, artificial intelligence, AI

## Abstract

**Background:**

Young adults experience stressors in their transition to adulthood and are at increased risk of mental ill-health. This risk is compounded by young adults’ low levels of mental health literacy and limited competencies in implementing strategies promoting mental health and well-being in their daily lives. Previous research suggests that digital mental health apps may be particularly effective in increasing the mental health literacy of young adults. In Sweden, there is a lack of research on young adults’ unique perspectives on what constitutes mental health, well-being, and ill-health—perspectives that could inform the coproduction of evidence-based interventions targeting these issues.

**Objective:**

The overarching aim of this study was to conduct a needs assessment as part of coproducing a digital mental health app for Swedish young adults. More specifically, the study addressed two research questions: (1) What do Swedish young adults perceive as contributing to the mental health, well-being, and ill-health of themselves and their peers? (2) What are Swedish young adults’ preferences and ideas on how a digital mental health app can support their mental health during young adulthood, including their perspectives on the app’s usability?

**Methods:**

We conducted semistructured interviews with 16 young adults and analyzed the data using reflexive thematic analysis.

**Results:**

Of the 16 study participants, 9 (56%) identified as women and 7 (44%) as men. Their mean age was 23.6 (SD 4.22; range 18-29) years. Furthermore, 56% (9/16) were pursuing or had obtained a higher education degree, while 44% (7/16) had completed or were in the process of completing a high school diploma. The interviews and subsequent analysis revealed three main themes: (1) “To feel that life is worth living”—pathways through pressures and pursuit of mental well-being during young adulthood, (2) “A personal space for working on one’s own mental well-being”—digital companionship with others, and (3) “Something that is designed for me”—customizing one’s digital mental health journey.

**Conclusions:**

In line with the preferences of Swedish young adults, the promotion of mental health and well-being through digital technology and eHealth should focus on a customizable app that supports balance in daily life while strengthening mental health competencies. The content should center on fostering and maintaining meaningful relationships and activities, addressing challenges such as negative social media use and stress recovery, and enhancing mental health knowledge and peer support. Future efforts should focus on researching young adults’ experiences of the life phase of emerging adulthood and its implications for mental health. In addition, future technical development and research on digital mental health apps should include the perspectives of stakeholders, such as mental health professionals, and involve prototype testing with diverse groups to ensure the app’s relevance, user engagement, and effectiveness.

## Introduction

### Background

Globally, young adults aged between 18 and 29 years disproportionally bear the burden of mental ill-health [1]. Young adults are at greater risk of being exposed to anxiety, stress, and depression compared to the general population [2]. As a result, 75% of individuals experiencing long-term mental ill-health report onset before the age of 24 years, affecting their transition to adulthood and limiting their participation in education, employment, and the community [3]. Furthermore, the World Health Organization [4,5] reports that suicide is the second leading cause of death among young adults. In Sweden, mental ill-health costs the Swedish economy approximately 5% of the gross domestic product [6], with young adults carrying a significant proportion of these costs. Research points to a mental health crisis among young adults, which is impacting national and global economies as well as welfare systems [3,7].

In this study, mental health is conceptualized as existing on a continuum ranging between mental ill-health and mental well-being [8]. This approach is favored over the more commonly used terms and discourse around mental illness, mental health conditions, or mental disorders [4]. This choice was made for 2 reasons: first, to align with the terminology guidelines set by the Swedish National Board of Health and Welfare; and second, because these terms are commonly used by Swedes to describe mental health on individual, group, and societal levels [8,9].

Swedish young adults report high levels of mental ill-health, driven by worries about their future, daily stressors, and challenges in balancing educational and employment demands [9]. This is not surprising, given the common difficulties associated with the phase of “emerging adulthood” [10]. Emerging adulthood as a developmental life stage encompasses young adults aged 18 to 29 years [9]. It is characterized by the challenges and opportunities in navigating new environments, establishing major life roles, and meeting new demands—often in the context of diminished social support and resources [9]. During this life stage, young adults describe experiencing feelings of uncertainty and being “in between,” struggling to choose between multiple life choices and possibilities [9]. Recent research [3,8] also points to the negative impact of new mega trends on the mental health of young adults, including concerns regarding climate change and the loss of biodiversity, increasingly toxic social media, and rising intergenerational inequalities. Increasingly, there is recognition [3] that young adults are experiencing an unprecedented mental health crisis and that there is a need for new insights and approaches.

In addition, negative community attitudes toward mental ill-health contribute to stigma and act as barriers to accessing support and mental health care [11]. Furthermore, limited community understanding of the signs signaling deteriorating mental ill-health and approaches to promoting well-being negatively impact the community’s ability to support young adults [12]. While there is a heightened awareness and normalization of mental ill-health among youth, driving an increasing demand for mental health services, this has not been paralleled by investment in mental health services [2,13]. Therefore, while there is increasing recognition of the stressors associated with young adulthood and the importance of this period in preventing long-term ill-health, young adults still infrequently receive or seek targeted support from mental health services [8,14].

Mental health promotion—supporting protective factors and healthy behaviors—has been proposed as a means of enhancing the mental health and well-being of young adults [13,15] and reducing the associated economic costs [7]. Advances in technology have enabled the development of digital health technologies and apps, which have emerged as key tools in promoting mental health across populations [16]. Digital tools have demonstrated particular efficacy in improving mental health literacy and supporting individuals with mental ill-health [17-19]. Mental health literacy, that is, the ability to recognize and manage challenges to one’s mental health [12], empowers individuals to adopt positive behaviors in daily life and support those around them [12]. At the community level, mental health literacy plays a critical role in preventing long-term mental ill-health, supporting suicide prevention efforts, reducing stigma, and promoting thriving communities [4]. Given their limited life experiences, many young adults commonly have particularly low levels of mental health literacy [8]. Globally, several digital mental health literacy interventions have targeted adolescents and young adults [18,19], demonstrating improvements in mental health outcomes through strengths-based approaches, peer support, life skills training, and increased resilience [19-21]. However, there is a lack of evidence-based digital mental health apps specifically tailored to the needs of Swedish young adults [22].

In summary, the stressors that young adults face in transitioning to adulthood can threaten their long-term mental health [3,9]. Young adults’ risk of mental ill-health is compounded by their generally low levels of mental health literacy, including their limited understanding of strategies promoting mental well-being in their daily lives [8]. Digital technologies, such as a mental health app, may provide a useful resource in delivering a strengths-based approach to mitigating mental health threats. Prior research highlights the importance of engaging stakeholders and coproduction in developing successful and culturally appropriate digital health interventions targeted toward the needs of a specific group [23-25]. However, less attention has been given to how young adults themselves perceive their everyday mental health challenges and specifically what kinds of digital support they find both relevant and accessible. Without this user-centered perspective, digital interventions risk missing the mark in addressing actual needs. Thus, there is a need for in-depth understanding of young adults’ experiences and preferences to inform the cocreation of more meaningful and effective digital mental health tools. In line with this thinking, this study aimed to explore Swedish young adults’ mental health needs during emerging adulthood, examining their perspectives on how digital technology could meet these needs, laying the foundation for the development of a culturally and contextually relevant digital mental health app. This approach is likely to enhance the efficacy, accessibility, and utility of a future digital mental health solution [24,26].

### Study Aim

The overarching aim of this study was to conduct a needs assessment as part of coproducing a digital mental health app for Swedish young adults. More specifically, the study addressed two research questions:

1. What do Swedish young adults perceive as contributing to the mental health, well-being, and ill-health of themselves and their peers?
2. What are Swedish young adults’ preferences and ideas on how a digital mental health app can support their mental health during young adulthood, including their perspectives on the app’s usability?

## Methods

### Study Context

The ultimate goal of this line of research is to coproduce an eHealth app supporting the mental health and well-being of Swedish young adults. The Medical Research Council’s framework for developing and evaluating complex interventions [27] and the design thinking approach [28] provide the theoretical foundation for this work, outlining the necessary steps in innovating new health care solutions—from inception to implementation. The initial phase, referred to as a “needs assessment” [27,28], involves understanding and defining the needs of the target audience of a proposed intervention as well as identifying the challenges and opportunities related to its successful implementation and use. Together, these approaches have demonstrated utility in coproducing innovative health promotion initiatives [29-31].

### Study Design

A qualitative descriptive interview study [32], guided by a formative research approach [33], was considered the most appropriate method to address this study’s aim and research questions. The formative research approach focuses on exploring a target group’s needs and preferences to ensure that a planned intervention is relevant, user-centered, and effective [33]. This research design has proven particularly useful in conducting needs assessments and informing eHealth interventions tailored to the specific needs of a defined target group [29,30]. In addition, this study is designed to align with the Standards for Reporting Qualitative Research [34], strengthening the study’s dependability and confirmability. The completed Standards for Reporting Qualitative Research checklist is available in Multimedia Appendix 1.

### Recruitment and Participants

A total of 16 participants were recruited via convenience and snowballing sampling [32] (Table 1). The number of participants considered adequate for a qualitative descriptive study is estimated to range from 15 to 20 [35]. Furthermore, previous studies with similar aims support the sufficiency of this number of participants [36,37]. The mean age of the participants was 23.6 (SD 4.22; range 18-29) years. They were literate in Swedish and actively using digital devices and apps.

**Table 1:** Sociodemographic characteristics of Swedish young adults participating in a qualitative study (March-September 2024) exploring mental health and preferences for digital mental health support.

Participant IDs	Gender	Age (years)	Educational level	Employment status or role
1	Man	28	High school diploma	Teaching assistant
2	Man	18	Ongoing high school diploma	Student
3	Man	28	University degree	Nurse in a psychiatric acute clinic
4	Man	25	University degree	Industrial technician
5	Woman	29	University degree	Occupational therapist at psychiatric rehabilitation unit
6	Woman	29	High school diploma	Care assistant
7	Woman	28	University degree	Employed at a municipality
8	Woman	18	Ongoing high school diploma	Student
9	Woman	20	High school diploma	Unemployed
10	Woman	18	Ongoing high school diploma	Student
11	Woman	26	Higher vocational education diploma	Video editor
12	Woman	22	Ongoing university degree	Student
13	Woman	25	Ongoing university degree	Student
14	Woman	24	University degree	Sustainability strategist
15	Man	18	High school diploma	Unemployed
16	Man	23	Ongoing university degree	Student

Convenience and snowballing sampling took place between March and September 2024. The first author (MKB) contacted 28 young adults who were distant acquaintances from past or current educational settings; workplaces; or sports clubs in southern, central, and northern Sweden. From a perspective of positionality, the author (MKB) had no ongoing contact or relationship with the young adults approached. Initial contact was made via SMS text messaging or a messaging app. The young adults were asked whether they were interested in participating or whether they could forward the invitation to someone else who met the inclusion criteria. If the young adult was interested, they were provided with an information letter outlining the key aspects of their participation. At the end of each interview, participants were asked whether they knew any peers who might be interested in taking part in the study.

### Data Collection

A semistructured interview guide (Multimedia Appendix 2) was developed in line with the study aims and research questions to ensure consistency across interviews [32]. The guide consisted of 2 focus areas: one eliciting the perspectives and experiences of the young adults related to mental health (eg, “Can you give examples of what mental health looks like among young adults in your community?”) and the other focusing on gathering the young adults’ preferences for the development of a digital mental health app (eg, “Can you give examples of important functionalities to make the digital mental health application usable?”)*.* Interviews loosely adhered to the interview guide, with broad, open-ended questions establishing the context, followed up with more specific, probing questions, ensuring a natural flow and an in-depth understanding of the topic. However, several interviews followed a more iterative style, with conversations naturally following participants’ views on the topic.

A pilot test of the interview guide was undertaken with 2 young adults who met the inclusion criteria, resulting in a simplification of the language, with the questions made more open-ended. Given that the pilot interviews provided valuable information relevant to the study’s aim, they were included in the data analysis. In line with participants’ preferences, interviews were conducted either digitally or in person by the first author (MKB) between March 17 and September 11, 2024. The interviews lasted an average of 48 (SD 7.09) minutes.

### Data Analysis

The interview transcripts were analyzed inductively, using reflexive thematic analysis, with themes emerging from the data [38]. This analysis method was chosen because of its emphasis on creating a narrative and story reflecting the study context and the voices of the participants [38]. Reflexive thematic analysis acknowledges researchers’ biases and accepts that the interpretation of the data is influenced by prior knowledge and experiences [38].

The ontological and epistemological position of the authors and this research is based on constructivism [39]. This approach assumes that knowledge is created relationally through humans interacting with each other in daily life [39]. The analysts (MKB, SG, BM, AL, and Moa Yngve, an external researcher [ER]) involved in this study have research roles and previous experience in conducting research within fields related to young adults’ mental health and well-being. Their broader research experience includes promoting community participation and functioning in daily life through a wide array of health promotion interventions. The first author (MKB) has personal experiences of mental ill-health during young adulthood. Both MKB and AL also have experience as close relatives of family members with long-term mental ill-health.

After transcribing the interviews verbatim, the first author (MKB) read the transcripts, familiarizing himself with the content. Although the interview guide questions were structured around 2 focus areas, the young adults’ answers were analyzed as a whole to capture overarching themes and patterns across the data. The initial 6 interviews were coded semantically, and a code chart reflecting the codes and initial themes from these interviews was created and developed iteratively and latently. Thereafter, using this chart, MKB coded the remaining interview transcripts. During the coding process, the code chart was revised and adapted several times as the understanding of overall themes emerged. The ER coded 2 interviews, providing feedback and suggestions for additional codes (MKB and the ER). Subsequently, the codes were mapped into clusters, which underpinned the themes (MKB, the ER, and AL). Across the data analysis process, the research team engaged in an iterative discussion of the coding and thematization until reaching a level of abstraction that was deemed logical and reflective of the material (MKB, SG, BM, AL, and the ER).

### Ethical Considerations

This research was approved by the Swedish Ethical Review Authority (2024-00582-01) and adhered to ethical principles outlined by the World Medical Association [40]. All participants provided written informed consent before the interviews and were informed of their right to withdraw from the study at any time. The collected data were deidentified, and only members of the research team involved in the analysis had access to the interview transcripts. No financial or other compensation was offered for participating.

As sharing one’s views on mental health can be sensitive and potentially evoke stress, anxiety, or worry, available supports were detailed in the information letter. In the event that participants experienced uncomfortable feelings or thoughts after engaging in the interviews, they were encouraged to contact the first (MKB) or last (AL) author for support—both have clinical experience in working with young people with mental health conditions. If this initial support was deemed insufficient by the young adult, given their consent, they would be referred to collaborating partners in mental health services. Sufficient time was allowed both before and after each interview as a strategy to promote rapport and create a safe space for sharing [41]. The sharing of an emotionally charged experience can bring meaning when participants deem the aims of a study worthwhile personally and to others [41]. Therefore, the overarching aims of the research project and its future implications for young adults’ mental well-being was emphasized in the information letter and before each interview started.

## Results

### Overview

**Figure 1:**
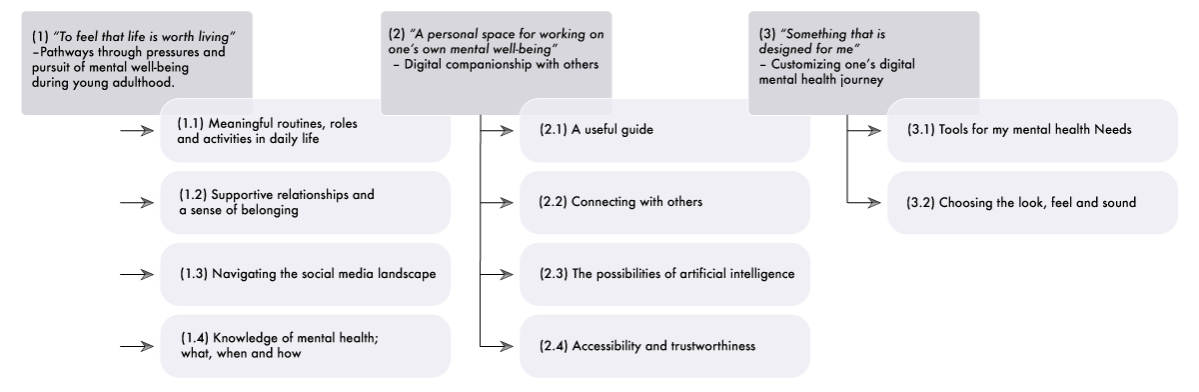
Themes and subthemes identified through thematic analysis of interviews with Swedish young adults (aged 18-29 years) between March and September 2024, reflecting their views on mental health and digital mental health support preferences.

The data analysis process revealed three themes: (1) “To feel that life is worth living”—pathways through pressures and pursuit of mental well-being during young adulthood, (2) “A personal space for working on one’s own mental well-being”—digital companionship with others, and (3) “Something that is designed for me”—customizing one’s digital mental health journey. The themes and subthemes are presented in Figure 1. Similarly, in Table 2, the themes and subthemes are illustrated with exemplar quotes, referred to as “illustrative examples,” along with condensed implications for designing the app.

**Table 2:** Overview of themes, subthemes, and supporting quotes derived from interviews with Swedish young adults, showcasing their implications for the design of a digital mental health app.

Themes and subthemes		Illustrative examples	Implications for the design of a digital mental health app
**1. “To feel that life is worth living”—pathways through pressures and pursuit of mental well-being during young adulthood**			
	1a. Meaningful routines, roles, and activities in daily life	“I hope it [the app] will allow people to reallocate their time so that, in some way, they can spend more time on what makes them feel good and on what is important to them.” [Study participant 14]	Include information about the importance of meaningful activities for mental well-being Support balancing activities that young adults need to perform with those that they want to perform
	1b. Supportive relationships and a sense of belonging	“I think that today a good safety net is probably essential for young adults to maintain mental well-being. We have so many factors, both societal and social, that easily lead to mental health issues. And that’s exactly why a very strong safety net, both socially and societally, is needed to support good mental health.” [Study participant 16]	Increase competencies in young adults collectively taking care of each other’s mental health
	1c. Navigating the social media landscape	“Just based on myself and others, I feel that on social media and the internet today, we are bombarded with so much information about these, what should we call them, elite people who might be just one percent of the population, and they are the only ones we see.” [Study participant 14]	Support young adults in navigating a balanced and healthy relationship with social media
	1d. Knowledge of mental health: what, when, and how	“My hypothesis is that we might have become a bit unaccustomed to talking about difficult feelings or tough moments. And that we could get better at showing our bad moments in public so people understand that life isn’t always good—you have your good times and your bad times. And by sharing, we can learn how to handle those tough moments and learn from each other.” [Study participant 16]	Provide young adults with tools to proactively increase knowledge about their mental well-being Teach young adults self-care and peer support strategies in managing mental health challenges Offer young adults direct access to additional mental health resources and support
**2. “A personal space for working on one’s own mental well-being”—digital companionship with others**			
	2a. A useful guide	“I think an app like this could be very helpful and supportive for those who are in this transition phase.” [Study participant 7]	The digital mental health app should be a guide to young adults’ mental well-being
	2b. Connecting with others	“A way to get to know other people and their experiences, to learn from them, and perhaps gain insights. Their experiences can provide support for our own lives and the situations we face.” [Study participant 15]	Provide safe ways for young adults to interact with others for mutual support and learning Facilitate the sharing of experiences to collectively address mental health needs
	2c. The possibilities of artificial intelligence	“I’ve also noticed, or heard, and even tested and experienced a pretty good effect from talking with AI [artificial intelligence], like ChatGPT.” [Study participant 3]	Cocreate an iterative, empathetic conversational AI agent to support mental health
	2d. Accessibility and trustworthiness	“If you turn to a tool that focuses specifically on mental health, you can expect it to meet that confidentiality standard that is essential for such conversations.” [Study participant 12]	Ensure that the app is available for free or offered at a minimal cost Maintain transparency in how personal data and their use are kept safe and secure
**3. “Something that is designed for me”—customizing one’s digital mental health journey**			
	3a. Tools for my mental health needs	“If someone wants a motivating app to mutually support themselves and others, they should be able to have that. And if someone just wants a listening, accepting app that doesn’t promote positive feelings, but is simply okay with everything, then maybe they should have that too.” [Study participant 11]	Offer customizable features to address diverse situations, challenges, and goals
	3b. Choosing the look, feel, and sound	“That you can choose what color you want on the outside of the app, or that the inside of the app allows you to select whether you want it to be pink or blue, and little things like that. I think it makes it easier to feel like, ‘This is my app.’” [Study participant 8]	Allow customization of the app’s appearance and interface to enhance use

### Theme 1: “To Feel That Life Is Worth Living”—Pathways Through Pressures and Pursuit of Mental Well-Being During Young Adulthood

#### Overview

This theme revolves around the needs identified as essential to nurturing the mental health and well-being of Swedish young adults. The participants in the study described mental health as a deeply personal experience, with an individual’s unique needs and preferences playing a crucial role in shaping their overall well-being. The importance of daily routines is captured in the first subtheme, (1a) meaningful routines, roles, and activities in daily life, highlighting the importance of nurturing mental well-being and growth as a young adult by actively doing what is important. In addition, the critical role of family, friends, and peers in supporting mental health was reflected in the second subtheme, (1b) supportive relationships and a sense of belonging, pointing to the importance of connectedness in everyday life. Furthermore, the study participants described contemporary societal phenomena as negatively impacting their mental well-being. This was mainly reflected in the third subtheme, (1c) navigating the social media landscape, with most of the study participants stressing the disempowering effect that social media has on their mental well-being and the experience of transitioning to adulthood. The fourth subtheme, (1d) knowledge of mental health: what, when, and how, captures participants’ experiences of inadequate mental health support during emerging adulthood, as well as their perception that they were not competent in actively promoting their own mental well-being.

#### Subtheme 1a: Meaningful Routines, Roles, and Activities in Daily Life

The young adults involved in this study fundamentally described “mental health” as a state of emotional well-being, enabling them to engage in personally meaningful activities and fulfill roles within their communities. Engaging in these activities and roles was seen as key in fostering a positive sense of self, essential in supporting participants’ transition to adulthood:

My personal experience...is that there have been things that...have been a bit tough. For example, not getting a job, or if I didn’t get a job, or things like getting a driver’s license.... There’s a pressure there because it feels like those are adult things one should be achieving in life. But by having things like training, going to the gym, playing floorball, and people to talk to, I’ve gained so much energy that even the things that have been heavy, difficult and full of anxiety to some extent have become easier.

Study participant 14

Being “responsible” and managing their activities of daily life, including getting sufficient sleep, exercising regularly, eating well, and adhering to healthy routines, were important to “staying on track” during challenging periods. Engaging in leisure activities “protected” against mental ill-health, while engaging in meaningful activities provided opportunities for exploring and embracing alternative roles, relieving their focus on careers and “succeeding.” Spending time on leisure activities enabled participants to develop and strengthen roles important for their relaxation, mental well-being, and rest, as well as support the growth of others.

However, several of the young adults described experiencing conflict in prioritizing leisure activities over work, study, or future-oriented obligations. Participants described themselves and their peers as focusing on meeting the necessary demands in achieving their career goals or “succeeding,” seeing this as negatively impacting their mental health.

#### Subtheme 1b: Supportive Relationships and a Sense of Belonging

Social support was key in sustaining participants’ mental health and aiding their recovery from the challenges stemming from their transition experiences. The availability of support from friends, family, and peers was seen as essential for both “surviving” and “living a good life”:

Relationships with people. Deep relationships with people. Being able to have people you can talk to and feel secure with. I would say that’s something important. It’s kind of one of those basic things, like food and water.

Study participant 14

By contrast, participants recognized feeling “isolated” and *“*withdrawing” from relationships, preferred activities, and their community as signs of mental ill-health:

I feel like if a friend, for example, is having a bad day, they can call or text and talk about it. But when things are genuinely tough in life right now, you don’t hear about it until afterward, when things are better again.

Study participant 15

#### Subtheme 1c: Navigating the Social Media Landscape

Social media use emerged as a complex factor influencing participants’ mental health. While it enabled connection with friends as well as access to inspiring influencers, an online community, and content that resonated with participants’ interests and sense of humor, social media also contributed to the feeling of being *“*locked in,” particularly in response to compulsive scrolling or “doom scrolling.” Excessive social media use resulted in participants feeling stressed, anxious, or “worthless,” as though they had “wasted” the day. A participant described the experience as follows*:*

If you feel like you haven’t done much in a day [and then spending time on social media], you end up feeling worse. You want to feel like you did something fun or something important with your day.

Study participant 12

Social media negatively impacted participants’ self-esteem, promoting narrow, “one-size-fits-all” ideals related to wealth, social status, and “successful” career paths. Instances of online harassment and exposure to unrealistic beauty ideals were described as further contributing to participants’ distress. While participants were aware of the potential harmful impact of excessive social media use, they reported struggling to reduce their engagement due to “fear of missing out.” This fear made disengaging from social media platforms particularly challenging. Several of the young adults expressed a specific desire for support and guidance on how to develop a healthier, more balanced relationship with social media. They emphasized the need for tools and strategies to mitigate its negative impacts while retaining its positive aspects:

The reason why many people feel bad today is probably also because of social media, I would say....There are influencers or other famous people, or maybe people who live in the same city as you, who you think look good. And then you want to be like them. And then when you don’t look like them maybe, or don’t wear makeup like they do, you feel like you don’t fit in. And then you can feel bad about it.

Study participant 8

#### Subtheme 1d: Knowledge of Mental Health—What, When, and How

Many participants described existing mental health care services as inadequate in meeting the diverse and evolving needs of young adults, leaving them disempowered in both managing their own mental health and supporting their peers. Participants further perceived that they lacked the knowledge to maintain their own mental well-being, which they felt contributed to their risk of mental ill-health:

If I take my own experiences, considering that I’ve dealt with mental health issues and still struggle with them from time to time, it took me four years to recognize the warning signs before I asked for help. When I first began, at the start of it all, I asked for help, but didn’t receive what I needed, and that made me feel like this was something to stay quiet about.

Study participant 10

During the interviews, the majority of the young adults provided more comprehensive responses about aspects of mental ill-health than about the positive, health-promoting factors associated with mental well-being:

Mental health...for some reason, sadly, one immediately thinks of mental ill-health.

Study participant 5

Most participants’ knowledge of mental health centered on noticing and responding to alarming symptoms in themselves and others. However, they expressed a strong desire to better understand mental health during emerging adulthood. Participants felt that collectively improving their understanding of approaches to fostering mental well-being in their daily lives could empower their generation.

### Theme 2: “A Personal Space for Working on One’s Own Mental Well-Being”—Digital Companionship With Others

#### Overview

This theme highlights young adults’ largely positive view of digital technology as a mental health support tool, as seen in the first subtheme, (2a) a useful guide. A digital mental health app held promise as a way to “actively put effort” into their well-being. The second subtheme, (2b) connecting with others—relates to the participants’ perception of a digital mental health app as a potentially valuable tool for fostering connections and increasing mental health knowledge. The third subtheme, (2c) the possibilities of artificial intelligence, explores the young adults’ views on artificial intelligence, while the fourth, (2d) accessibility and trustworthiness, expands on data security and access to a digital mental health app.

#### Subtheme 2a: A Useful Guide

Many young adults expressed a strong desire to learn more about maintaining mental health and developing knowledge of how mental ill-health is manifested, coping mechanisms, and routines promoting well-being. However, they described feeling frequently overwhelmed by the vast amount of information available online and struggling to navigate and curate it. They envisioned a mental health app as offering clear guidance through an intuitive interface, making it easier to browse relevant topics, categories, and information relating to mental health:

Perhaps guides on how to proceed in order to somehow succeed in getting out of a dip. Even though it’s strange maybe to say “just do it”...But if you don't have it on paper, it's often very difficult to see that you can get there. I think the opportunity to get a clear picture of how things can be handled and how one can get help, simply.

Study participant 7

Participants stressed the need for an app that offers practical advice for “handling daily life,” helping to bridge the gap between their desire for knowledge and their difficulty in finding and prioritizing the right information online. A digital guide tailored to young adults’ mental health challenges was seen as a promising tool for navigating the life phase of emerging adulthood.

Despite the aforementioned features and optimism, some participants questioned whether a digital app could effectively support the mental well-being of young adults; for instance, some viewed the current digitalization of society and health care services as “ineffective” and disturbing due to their belief that therapy provided in person is “better” and more “humane.”

#### Subtheme 2b: Connecting With Others

A digital solution focusing on enabling interactions between young adults and providing a forum for them to discuss their mental health needs was viewed positively:

I also think it’s important for an application like this to have some kind of forum that promotes interaction, whether it’s between users or between a moderator and users. I think that would be helpful. We need more platforms to talk about this in safe spaces.

Study participant 16

Existing forums were frequently mentioned and described as providing a daily space to discuss worries or questions, as well as to gain insights and competencies. However, participants noted the importance of ensuring a safe space for discussing mental health and ill-health, while maintaining openness, but they cautioned that an open forum could be misused or abused. Participants noted that peer support could take the form of inspirational quotes as well as stories and narratives showcasing elements of “recovery” and “meaningfulness” in daily life despite crises, injuries, or chronic disease:

For me it has meant a lot to see people who may have experienced crises or changes in life but who have found other paths.

Study participant 9

#### Subtheme 2c: The Possibilities of Artificial Intelligence

Participants frequently identified opportunities for integrating artificial intelligence into the app, imagining an empathetic conversational chatbot capable of helping users process difficult thoughts and feelings through both voice and text. The young adults shared their expectations and hopes for a cutting-edge technological solution (eg, ChatGPT) that would provide a seamless and fully responsive user experience:

It should be easy speaking with “it,” and that you don’t have to correct your phrases all the time. That it understands you while you speak. That it just doesn’t straight up read a website from “1177” [Sweden’s national health care guide, offering medical information and digital services]. The information you receive should be adjusted and align specifically with the challenge described, and only information related to what you've already told.

Study participant 3

#### Subtheme 2d: Accessibility and Trustworthiness

Another potential concern voiced by all participants was whether it would cost money to use the app. They stressed the importance of making it free of charge, or at most, available for a small fee. Data security was expressed as a key concern, with participants questioning where and by whom confidential content could be accessed or potentially misused. Transparency regarding the measures taken to ensure data security and confidentiality was seen as of high importance for building trust from future users:

I think being honest with the user is the most important thing. So that people feel like individuals and not just one in the crowd.

Study participant 12

Some of the young adults described their hesitation in using existing digital mental health apps, citing uncertainty or lack of clarity about the companies behind them. They were instinctively more positive and trusting toward digital mental health apps created and offered by public health care agencies or universities.

### Theme 3: “Something That Is Designed for Me”—Customizing One’s Digital Mental Health Journey

#### Overview

This theme emphasizes the critical importance of enabling users to tailor the app to their personal needs, preferences, and wishes. The first subtheme, (3a) tools for my mental health needs, pertains to customizable functionality and features that support each individual’s unique mental health needs and aspirations. The second subtheme, (3b) choosing the look, feel, and sound, reflects the young adults’ desire to control how the digital mental health app interacts with them, including customization of its appearance, tone, and user experience.

#### Subtheme 3a: Tools for My Mental Health Needs

Ultimately, the study participants envisioned a digital mental health app as not only providing mental health support but also empowering them to navigate the complexities of transitioning to adulthood:

The right way to handle one’s problem—based on one’s own circumstances and issues. And what feels best, what fits best at the moment.

Study participant 3

By incorporating peer support, personalized recommendations, and artificial intelligence–driven technology, the app could potentially serve as a hub for addressing the mental health needs of young adults:

I have a lot of anxiety when I have to go out, but last time I did it, I called and talked to someone, and it worked. I can try that again.

Study participant 4

Some of the young adults highlighted the potential benefits of using digital technology to structure daily activities, manage time, and balance priorities. Examples included checklists; tools for tracking health and activity patterns; and common calendar functions such as reminders and visualizing schedules over days, weeks, or months. Similarly, many of the young adults expressed a desire to ideate, display, track, and follow up on personally meaningful mental well-being goals over time. Gamification elements, such as leveling up or receiving rewards, were also suggested as effective mechanisms to encourage continued use and motivate young adults to pursue their mental health goals.

#### Subtheme 3b: Choosing the Look, Feel, and Sound

Customization emerged as a key design feature, with participants wanting the app to feel uniquely tailored to them. They requested customizable features such as color schemes, interface layouts, and content preferences; for example, they wanted options for receiving information in various formats—text, audio, or video—based on individual learning styles. Some even suggested options to choose the voice reading information:

I remember that when I was feeling very low when I was younger, at my worst moments of anxiety, I couldn’t even read. There was just too much swirling around in my head, so I would read the words, but nothing would actually sink in. In those situations, it was always easier for me to listen to something.

Study participant 9

## Discussion

### Principal Findings

#### Overview

To the best of our knowledge, this is the first study in Sweden to conduct a needs assessment with young adults as part of coproducing a digital mental health app. In summary, young adults emphasized the importance of nurturing meaningful relationships and activities to support their mental health and well-being. Participants identified several key contributors to mental ill-health during emerging adulthood, including negative social media use, societal pressures, occupational imbalance, and difficulties in managing and recovering from stress and anxiety. They also expressed a positive outlook toward a tailored app addressing these challenges, highlighting its potential to enhance mental health literacy, foster peer support, and serve as a tool for achieving a better balance between what they “wanted to do” and “needed to do.” Customization, both in terms of visual design and functional features, was considered essential for fostering user engagement and achieving effective outcomes. The study’s 2 guiding research questions structure the discussion in the following subsections.

#### Swedish Young Adults’ Views on the Causes of Mental Health, Well-Being, and Ill-Health

The findings of this study highlight young adults’ perspectives on mental health and ill-health, which result from the complex interplay between individuals, groups, and sociocultural phenomena. First, the participants essentially viewed mental health and well-being as a state of being—characterized by the ability to engage in personally meaningful activities and to balance what they wanted to do with what they needed to do in daily life. Furthermore, the findings show that young adults associate mental well-being with creating room for leisure activities and engaging in activities that bring them joy amid stressful career- and success-dominated lives. However, the young adults often struggled with the skills and competencies needed to prioritize these types of activities over work or study obligations.

Research examining young adults’ mental health and well-being points to the importance of meaningful routines, habits, and activities as protective factors against mental ill-health [16,42,43] and essential in developing and cultivating a sense of identity as an adult [8,9]. The findings of this study align with the concept of “occupational balance” [44] from the fields of occupational science and therapy, which refers to an individual’s experience of maintaining a sufficiently varied and meaningful range of occupations and activities in daily life [45]. Research in this field has shown positive relationships between occupational balance, quality of life, and mental well-being [45,46]. Despite these insights, there is a notable lack of research specifically examining the components of occupational balance among young adults, particularly during emerging adulthood. More qualitative and quantitative research is needed to better understand their experiences and needs at a population level. Given the challenge that emerging adulthood poses to established routines and roles [9], promoting occupational balance through digital tools may offer an untapped opportunity to support young adults in managing their stress, enhancing their life satisfaction, and fostering their identity development.

The findings reveal 2 primary contributors to mental ill-health among young adults. The first is the adverse effects of social media use, which fosters unhealthy comparisons, negative self-image, and self-doubt regarding life choices. The adverse effects of social media use on the study participants’ mental ill-health were mainly exacerbated by “doom scrolling” [47], a behavior characterized by compulsive engagement with distressing content. This habit often leads to feelings of entrapment, frustration, and involuntary time loss. Participants often described a cycle of overuse of social media despite recognizing its negative consequences, underscoring the addictive nature of social media and its impact on mental well-being. These patterns align with existing research, which consistently links excessive social media use to heightened psychological distress and a diminished sense of self-worth [48-50]. Of note was the finding that young adults also experienced social media positively. For some, it provided inspiration, connections, and a source of humor in their daily life. Consistent with previous research, our findings highlight the complex meaning and effects of engaging with social media and its impact on the mental health of young adults [51,52]. Collectively, these findings suggest that any future digital mental health app aiming to support the mental health of young adults should focus on helping them to develop a more healthy, conscious relationship with social media through knowledge, competencies, and peer support.

The second contributor to the mental ill-health of the young adults in this study was the challenge of navigating emerging adulthood amid increasing societal uncertainty. The young adults highlighted the connection of societal and sociocultural uncertainty with increased mental ill-health, a phenomenon that can be understood through the concept of “liquid modernity” proposed by Bauman [53]. In this fluid and ever-changing societal landscape, traditional markers of adulthood—such as financial independence, home ownership, and career stability—have become increasingly elusive, exacerbating anxiety, insecurity, and mental ill-health among young adults [3,9,53]. This is driven by economic inequalities, a challenging labor market, digitalization, global crises, and shifting social norms, leaving young adults uncertain about their futures [13]. Young adults report increasing experiences of inadequacy, isolation, and self-doubt among themselves and their peers [3,9]. The expectation to succeed in a world of constant change heightens the risks of burnout from stress, anxiety disorders, and depression. The study participants expressed feeling unprepared for these challenges, grappling with a lack of knowledge and competencies as well as insufficient mental health support at institutional or societal levels that is tailored to their generation’s needs. To counter these rapid and often pathogenic societal developments, increasing the individual and group resilience of young adults through efforts to bolster their mental health and well-being may be a viable and impactful solution.

#### Swedish Young Adults’ Preferences and Thoughts on Digital Mental Health Support and Usability

Conducting a needs assessment to research and incorporate the preferences, needs, and suggestions of future users is recommended as a key step in developing eHealth innovations [27]. This approach not only enhances user engagement, acceptability, and use but also helps to identify critical factors for successful implementation [26].

A key finding of this study was that the young adults were positive toward using digital technology as a self-support tool for mental well-being and the prevention of mental ill-health. Similar attitudes and beliefs have been reported in recent studies [54,55]. Previous research [56-58] has demonstrated the efficacy of online tools in enhancing young adults’ awareness and competencies in proactively promoting mental health and responding to early signs of worsening mental ill-health. However, most eHealth-based mental health literacy interventions have primarily targeted individuals already experiencing mental ill-health, with an underrepresentation of initiatives developed to strengthening and improving mental well-being and resilience [56]. Thus, there are strong incentives for supporting young adults to handle the stressful life phase of emerging adulthood. Health-promoting efforts can play a vital role in improving the daily lives of young adults in Sweden, thereby helping to prevent suicides and distress as consequences of mental ill-health.

Another key finding of this study points to the importance of enabling personal customization in the functionality and appearance of the digital mental health app. Inviting users to choose from a variety of options – for instance, color schemes, media formats, screen content, or areas of mental health – can significantly increase the likelihood of sustained engagement among young adults. Prior studies support the interactions between customization, user empowerment, and use [59-61]. These findings underscore the need for the digital mental health app to incorporate customization features wherever technologically feasible.

Another mode of customization highlighted by the participating young adults was the use of intuitive and flexible generative artificial intelligence—particularly an empathetic chatbot—to meet their specific mental health literacy needs. Research [62,63] has shown that chatbots can be acceptable and efficacious in supporting mental health, provided they make accurate assumptions and personalize the interactions with each user. However, significant limitations of this technology remain, particularly in adequately understanding and addressing mental health crises, as well as in navigating ethical and privacy concerns [62,63]. Apps that use artificial intelligence should be cautiously coproduced with young adults to harness its capabilities to deliver a personalized user experience, simultaneously accounting for its limitations and associated risks.

Access to peer support through videos, meetings, or forums was identified as a central feature to be built in for inspiration, destigmatization, learning, and a holistic understanding of mental health. Previous research on implemented digital mental health apps has shown them to be effective in providing empowering peer support [64]. One important factor for successful implementation is balancing a moderated safe space with openness to discuss diverse topics [65]. Therefore, the incorporation of feasible and secure software features to enable meaningful and safe peer support should be prioritized in the development of the digital mental health app.

Another key finding of this study is the appreciation young adults expressed when seeing peers share their paths to recovery on social media. Such narratives had a positive impact on their own sense of hopefulness and connectedness. A previous study [66] similarly demonstrated the positive effects of using others’ recorded recoveries from mental ill-health to strengthen one’s sense of hopefulness and connectedness. In the article “The Lancet Psychiatry Commission on Youth Mental Health,” McGorry et al [3] emphasize the importance of viewing emerging adulthood through the lens of the “hero’s journey.” This perspective frames this life phase as a period of normal struggle and adventure with the goal of gaining maturity and exploring the world, coupled with a “return” or “homecoming” where individuals support and guide others on similar paths. Applying this perspective to mental health challenges and periods of ill-health can, according to the authors, serve to normalize experiences, foster empowerment, and reduce stigma [3]. Other research further supports this approach, finding that the “hero’s journey” perspective can increase an individual’s sense of agency and provide deeper meaning in life [67]. Therefore, providing stories of other young adults’ recovery journeys in the digital mental health app could be valuable for enhancing the mental well-being of young adults.

While the young adults generally perceived a digital mental health app as accessible and promising in meeting their needs, notable caveats exist. Study participants identified risks related to online security and the potential leakage of private information as potential barriers to active use. Transparency regarding how users’ data are stored and protected from misuse was emphasized as essential for building trust among young adults.

These findings align with previous research [54,55]. Therefore, to address these concerns, it is crucial to carefully choose a secure storage solution for users’ confidential information and to actively communicate how their data are managed and safeguarded.

### Strengths and Limitations

In this study, collecting data via semistructured interviews enabled participants’ experiences and perspectives to be explored in depth [32]. Furthermore, a clear description of the sociodemographic backgrounds of participants, including age, educational attainment, and employment status, informs judgments about the transferability of the findings [68] and exemplifies the contemporary and diverse life circumstances of Swedish young adults. We believe that the transferability of the findings is further supported by the broad inclusion criteria adopted in this study, which resulted in a diverse sample of Swedish-speaking young adults aged 18 to 29 years who used digital devices daily.

The inclusion of quotes and exemplars representing the majority of study participants further strengthens the trustworthiness of the study, as recommended by Korstjens and Moser [68]. In addition, the credibility of our analysis and results is reinforced by the reflexivity statement presented in the Methods section [35,38], which details the theoretical assumptions underpinning the data analysis and acknowledges the authors’ positionality. In regard to this study, the coding and generation of themes were influenced by both the analysts’ prior experiences of conducting research in the field of young adults’ mental health and their personal or familial experiences of mental ill-health. This may in turn bolster the credibility [68] of the study findings.

The confirmability of the study was further strengthened by the involvement of 3 analysts (MKB, AL, and the ER) in the data analysis process, including reading and discussing the interview transcripts, and with all analysts (MKB, AL, SG, the ER, and BM) actively engaged in developing the codes and themes and subthemes, as well as participating in debriefing sessions. Overall, these processes of intersubjective interpretation during data analysis [38,68] contributed to enhancing the study’s credibility.

The findings of the study are limited by the absence of member checking of the transcripts or final analysis with participants, as recommended by Korstjens and Moser [68]. While the first author (MKB) reflected deeply during data collection and analysis, these thoughts were not captured in a reflexive journal [38,68] during the research process, detracting from the confirmability of the findings. Finally, the conformability and neutral framing [68] of the research data may have been weakened by the analysts’ prior subject experience and knowledge.

### Conclusions

This needs assessment provides valuable insights for coproducing digital mental health apps tailored to young adults. In line with the preferences of Swedish young adults, the promotion of mental health and well-being through digital technology and eHealth should focus on a customizable app that supports balance in daily life while strengthening mental health competencies. The content should center on fostering and maintaining meaningful relationships and activities, addressing challenges such as negative social media use and stress recovery, and enhancing mental health knowledge and peer support. Future efforts should focus on researching young adults’ experiences of the life phase of emerging adulthood and its implications for mental health. In addition, future technical development and research on digital mental health apps should include the perspectives of stakeholders, such as mental health professionals, and involve prototype testing with diverse groups to ensure the app’s relevance, user engagement, and effectiveness.

## Appendix

Multimedia Appendix 1 Standards for Reporting Qualitative Research (SRQR) checklist.

Multimedia Appendix 2 Interview guide for data collection between March and September 2024.

## Abbreviations

ER: external researcher

## Data Availability

Due to the sensitivity of research on young adults’ mental health, deidentified interview transcripts are available from the corresponding author on reasonable request.
