# Supplementary material for: Needs and Preferences of Swedish Young Adults for a Digital App Promoting Mental Health Literacy, Occupational Balance, and Peer Support: Qualitative Interview Study
Source: JMIR Form Res. 2025 May 23;9:e71563. doi: 10.2196/71563 (PMC12144469; doi:10.2196/71563)
Supplement: Multimedia Appendix 2 [file formative_v9i1e71563_app2.docx]

#
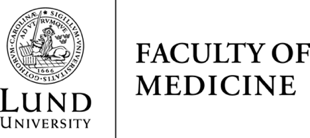


Needs and Preferences for Digitally Promoting Mental Health Literacy, Occupational Balance, and Peer Support among Swedish Young Adults:
A Qualitative Interview Study

Martin Karaba Bäckström, Sonya Girdler, Ben Milbourn, Annika Lexén

**Interview Guide**

Have the participant read the information letter and provide consent.

Provide a framework for the interview: Estimated time around 45 minutes, inform them that the conversation will be recorded, and mention the option to withdraw participation at any time.

Brief description of the larger research project and the development of an e-health solution for young adults' mental health.

**Participant Information:**

- Participant's name
- Participant's age
- Participant's gender identity
- Participant's level of education
- Participant's current occupation

| **Question Areas/Main Questions** | **Follow-up Questions/Prompts** |
| --- | --- |
| *Perspectives on Mental Health and Digital Tools for Health* |  |
| 1. What is mental health? 2. What contributes to young adults feeling well? 3. What is mental ill-health? 4. What contributes to young adults feeling unwell? 5. How do you use websites/apps for your well-being? 6. Do you think young adults would benefit from an application providing support for mental health? | a) How does mental ill-health present itself in young adults around you? b) What contributes to young adults feeling well? c) Why do you use these apps? Why do you like them? d) In what way? For whom? How? |
| *Perceptions of Delivered Intervention* |  |
| 1. What do you think an application promoting mental health in everyday life should contain to meet the needs and preferences of young adults? | a) Relate to the examples of poor well-being/causes of mental ill-health.  b) Connect to what they mentioned as factors that promote mental health. |
| *Promoting Factors for using the Intervention* |  |
| 1. What opportunities do you see for an application to promote mental health in the daily lives of young adults? 2. What would make the application easy to use? 3. What is important for young adults to want to use the application? 4. What would such a solution need to look like to be appealing to you? | a) What, in your opinion, would be signs that the application supports young adults' mental health in everyday life?  b) Can you specify which features would make the application easy to use?  c) What visual elements would make the application appealing (color, design, imagery)? |
| *Hindering Factors for using the Intervention* |  |
| 1. What barriers do you see that might prevent people from using the application? 2. What would make someone not want to use the application? | 1. Can you specify which features would make the application difficult to use? 2. What visual elements would make the application unappealing (color, design, imagery)? |
| *Future Involvement* |  |
| 1. Would you be interested in helping or being involved in developing the application? 2. May I contact you again if I have further questions or reflections regarding your responses from this interview? | (Tell the study participant about the design process for developing the application). |
| *Snowball Sampling* |  |
| 1. Do you have any suggestions for others we could speak with to better understand young adults' thoughts on applications and mental health? |  |
